# Author Correction: Paramagnons and high-temperature superconductivity in a model family of cuprates

**DOI:** 10.1038/s41467-023-38825-7

**Published:** 2023-05-25

**Authors:** Lichen Wang, Guanhong He, Zichen Yang, Mirian Garcia-Fernandez, Abhishek Nag, Kejin Zhou, Matteo Minola, Matthieu Le Tacon, Bernhard Keimer, Yingying Peng, Yuan Li

**Affiliations:** 1 International Centre for Quantum Materials, School of Physics, Peking University, Beijing, 100871 China; 2 Max Planck Institute for Solid State Research, Stuttgart, 70569 Germany; 3 Diamond Light Source, Harwell Science & Innovation Campus, Didcot, Oxfordshire OX11 0DE UK; 4 Institute for Quantum Materials and Technologies, Karlsruhe Institute of Technology, Karlsruhe, 76133 Germany; 5 Collaborative Innovation Centre of Quantum Matter, Beijing, 100871 China

**Keywords:** Superconducting properties and materials, Magnetic properties and materials

Correction to: *Nature Communications* 10.1038/s41467-022-30918-z, published online 07 June 2022

The original version of this Article contained an error in Fig. 2, in which the vertical energy axes of panels **b** and **d** were scaled by 122% relative to panels **a** and **c** instead of the 130% stated in the caption. This figure has been corrected in both the PDF and HTML versions of the article.

The original version of this Article contained an error in the fifth sentence of the section ‘Raman scattering result’, which incorrectly read ‘Near-optimal doping, 2Δ_SC_ is known to be about 86 meV (ref. 18, compared to 93 meV in our sample) for Hg1201.’ The correct version states ‘ref. 30’ in place of ‘ref. 18’. This has been corrected in both the PDF and HTML versions of the Article.

The original version of this article contained an error in the fourth from the last sentence of the section ‘Analysis of RIXS spectra’ of the Methods, which incorrectly read ‘This result is generally consistent with previous RIXS results for doped cuprates, e.g., in ref. 40.’ The correct version states ‘ref. 8’ in place of ‘ref. 40’. This has been corrected in both the PDF and HTML versions of the Article.

The original version of this article contained an error in Eq. (5), which incorrectly read:

$$\omega (H)=2J\sqrt{\frac{1-{(\cos (2\pi H)+1)}^{2}}{4}}.$$

The correct form of Eq. (5) is:

$$\omega (H)=2J\sqrt{1-\frac{{(\cos (2\pi H)+1)}^{2}}{4}}.$$

This has been corrected in the PDF and HTML versions of the article.

The original version of the Supplementary Information associated with this article contained errors in the reference numbers. The HTML has been updated to include a corrected version of the Supplementary Information.

## Supplementary information

Updated supplementary information

