# Supplementary material for: Paramagnons and high-temperature superconductivity in a model family of cuprates
Source: Nat Commun. 2022 Jun 7;13:3163. doi: 10.1038/s41467-022-30918-z (PMC9174205; doi:10.1038/s41467-022-30918-z)
Supplement: Supplementary file 1 — Supplementary Information [file 41467_2022_30918_MOESM1_ESM.pdf]

Supplementary Information

Paramagnons and high-temperature superconductivity in a model family of cuprates

Lichen Wang[1,2,#], Guanhong He[1,#], Zichen Yang[2], Mirian Garcia-Fernandez[3], Abhishek Nag[3], Kejin Zhou[3], Matteo Minola[2], Matthieu Le Tacon[4], Bernhard Keimer[2], Yingying Peng[1,5,*], Yuan Li[1,5,*]

[1]International Centre for Quantum Materials, School of Physics, Peking University, Beijing 100871, China
[2]Max Planck Institute for Solid State Research, Stuttgart 70569, Germany
[3]Diamond Light Source, Harwell Science & Innovation Campus, Didcot, Oxfordshire OX11 0DE, United Kingdom
[4]Institute for Quantum Materials and Technologies, Karlsruhe Institute of Technology, Karlsruhe 76133, Germany
[5]Collaborative Innovation Centre of Quantum Matter, Beijing 100871, China
[#]These authors contributed equally to this work.
*yingying.peng@pku.edu.cn (Y.P.); yuan.li@pku.edu.cn (Y.L.)

This file includes:

Supplementary Figures 1 to 13

Supplementary Tables 1 to 5

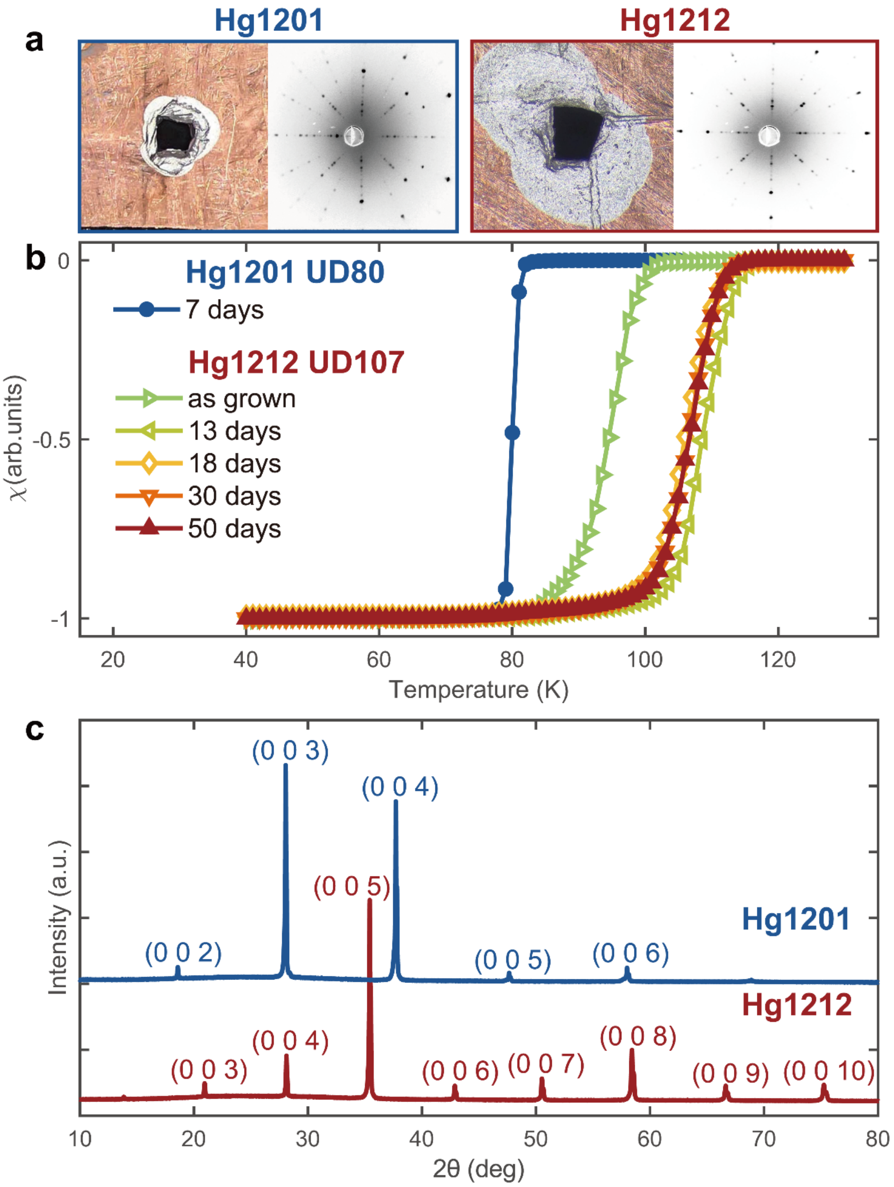

Supplementary Figure 1. Hg1201 and Hg1212 crystals used in this study. a, Photos of our Hg1201 and Hg1212 crystals mounted on a copper sample holder, before the RIXS experiments. X-ray Laue images taken on natural surfaces of Hg1201 and Hg1212 single crystals are displayed together. **b,** Magnetic susceptibility measurements of our crystals after annealing in air at 480 °C for the indicated amounts of time. The measurements were performed upon warming the crystals with a magnetic field of 5 Oe applied along the *c* axis, after cooling the crystals in zero field. T_c is determined from the midpoint of the transitions to be 80 K and 107 K for Hg1201 and Hg1212, respectively, at the end of the annealing. **c,** X-ray diffraction data taken on single crystals of Hg1201 and Hg1212, with the momentum transfer along the *c* axis. The *c*-axis lattice constants are determined to be 9.53 Å and 12.63 Å at room temperature, respectively.

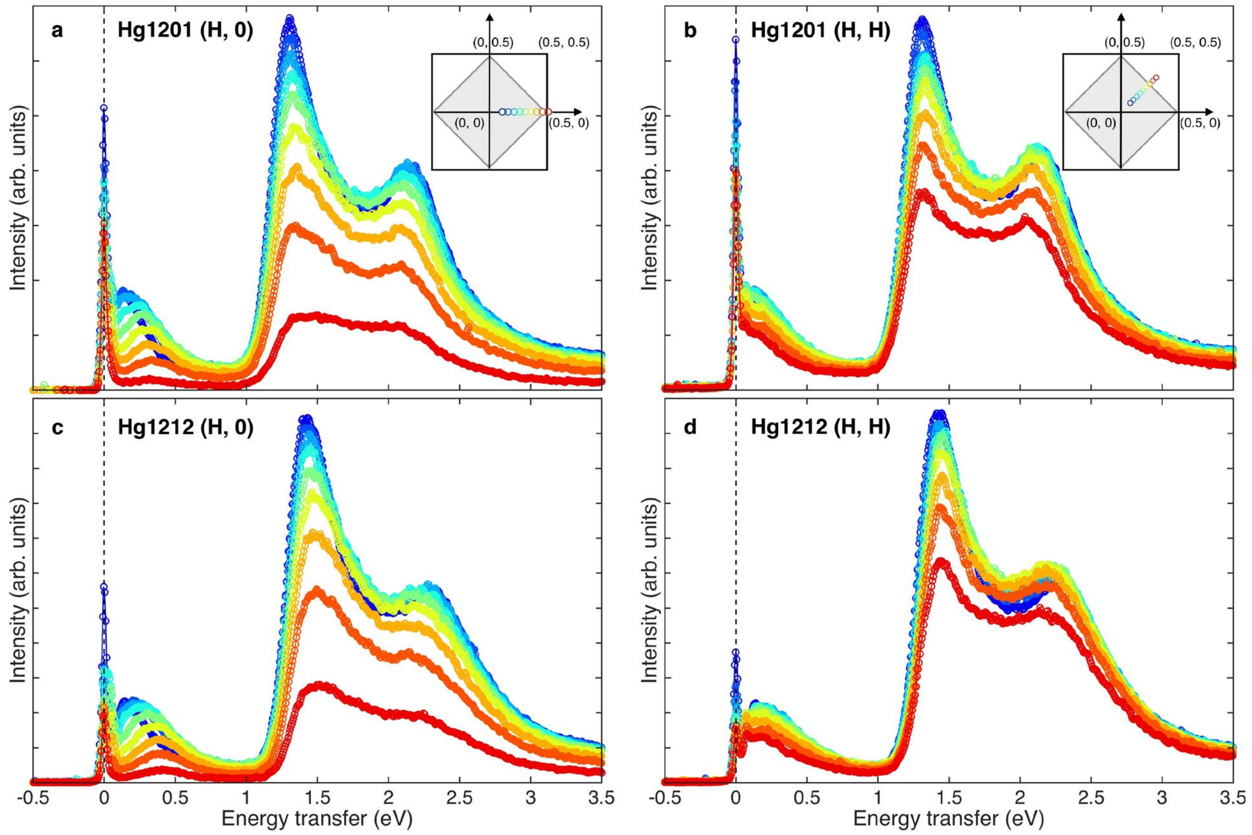

Supplementary Figure 2. Raw RIXS spectra including *dd* excitations. a-b, Raw spectra measured at $\mathbf{Q}_{//}$ = (H, 0) and (H, H) for Hg1201, respectively. **c-d,** Raw spectra for Hg1212. Insets indicate the in-plane momentum trajectories, colour-coded with the data points; grey area is the first magnetic Brillouin zone.

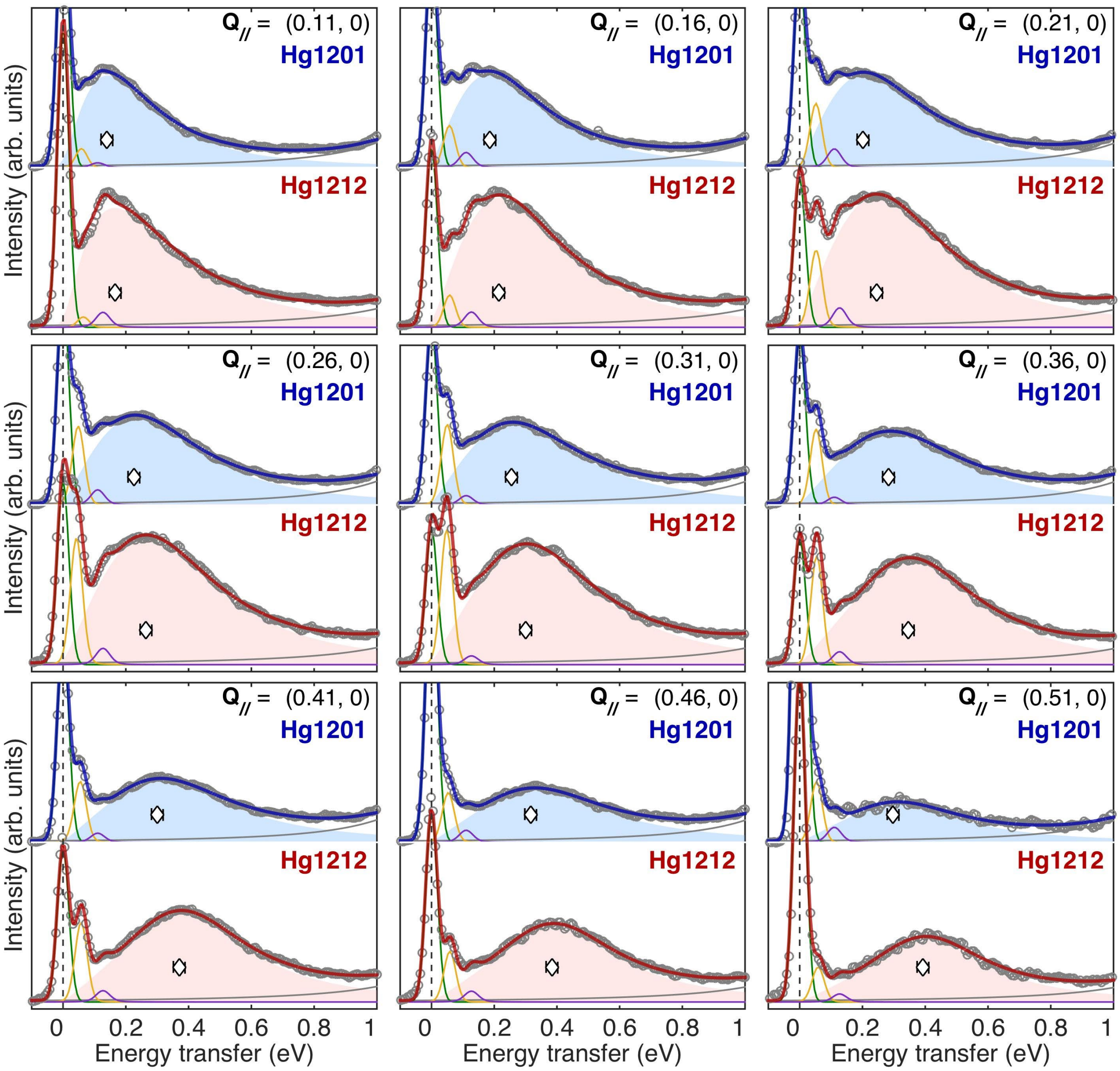

Supplementary Figure 3. Q-by-Q comparison and decomposition of RIXS spectra along (*H*, 0). Empty circles are measurement data points. The spectra are decomposed into a sum of an elastic peak (green), a single-phonon peak (yellow), a two-phonon peak (magenta), a paramagnon peak (shaded area), and a weakly energy-dependent background (grey). Blue and red solid lines are the sum of all best-fit components for Hg1201 and Hg1212, respectively. Diamond symbols indicate the energy position of the paramagnon peak's maximum, error bar representing the uncertainty of the estimate (1 s.d.). Vertical dashed line marks the zero energy, which is set by the fit result of the elastic peak position. Summary of fitting parameters for the paramagnon component is presented in Supplementary Table 1.

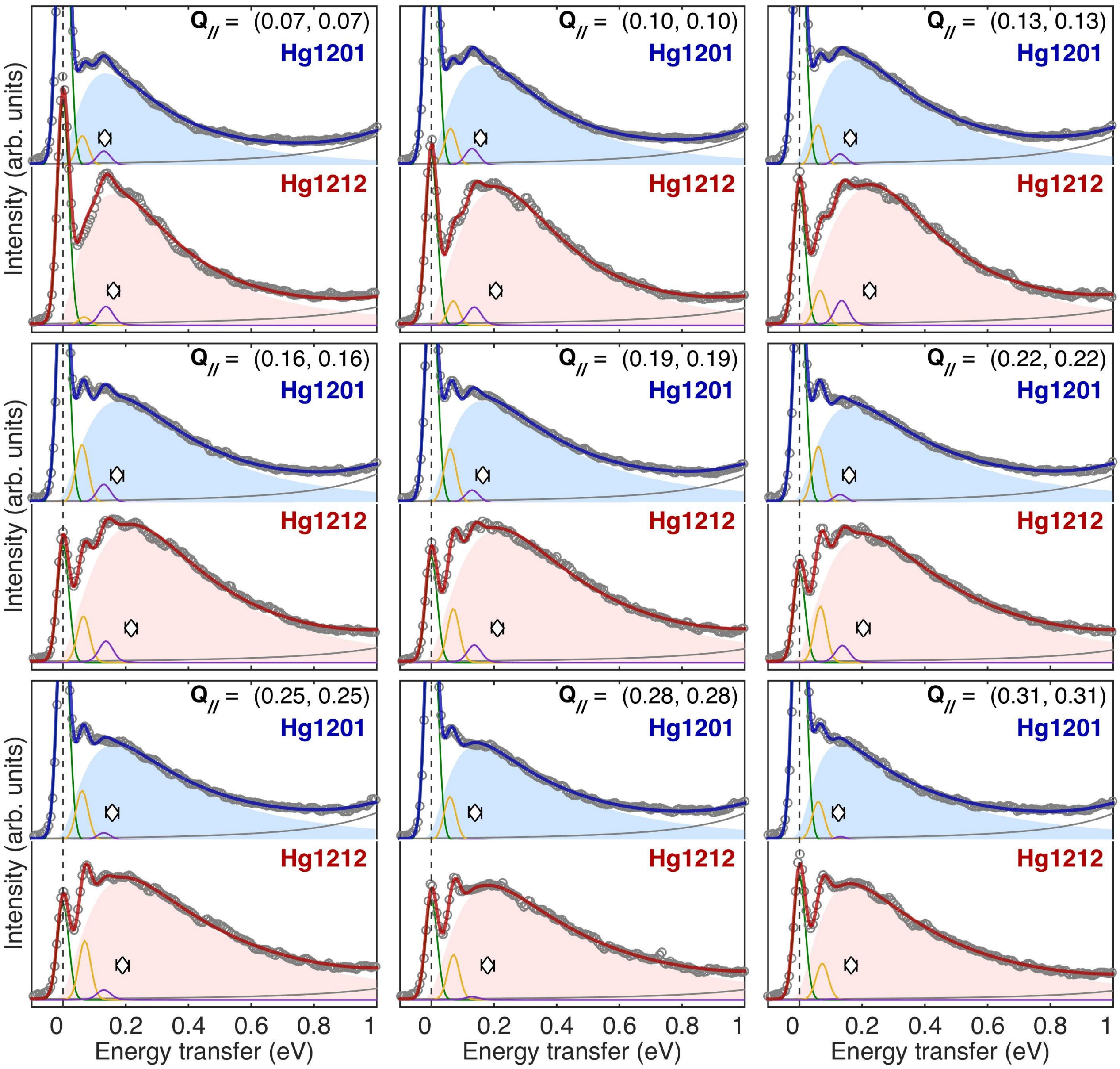

Supplementary Figure 4. Q-by-Q comparison and decomposition of RIXS spectra along (*H*, *H*). See captions of Supplementary Figure 3.

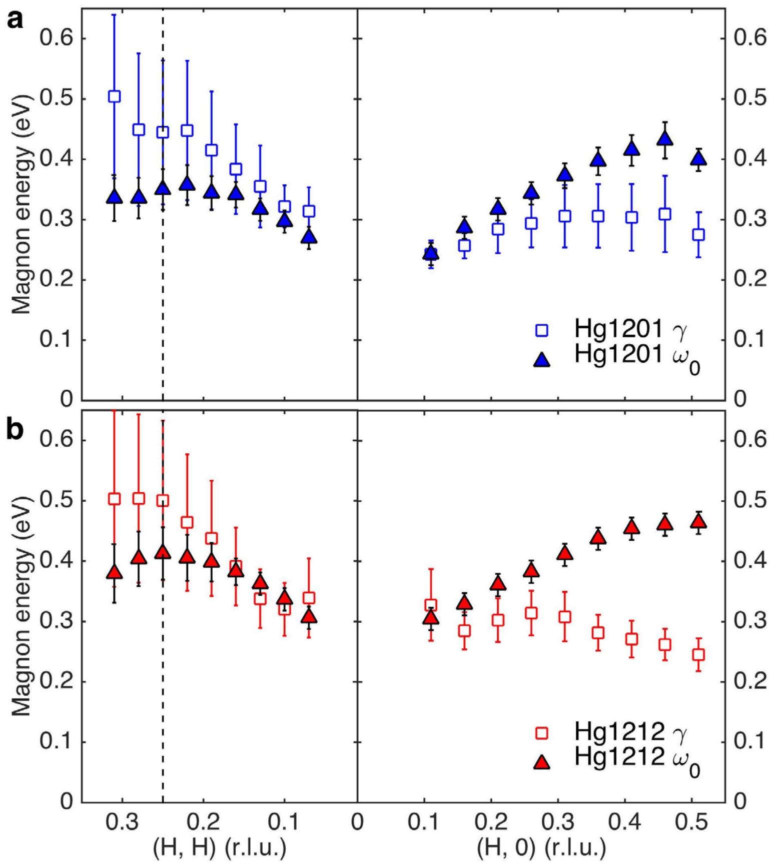

Supplementary Figure 5. The undamped energy ω_0 and the damping γ for Hg1201 and Hg1212. Vertical dashed line indicates magnetic zone boundary along (*H*, *H*). Error bars indicate uncertainty of the fitting estimate (1 s.d.), see Methods for details.

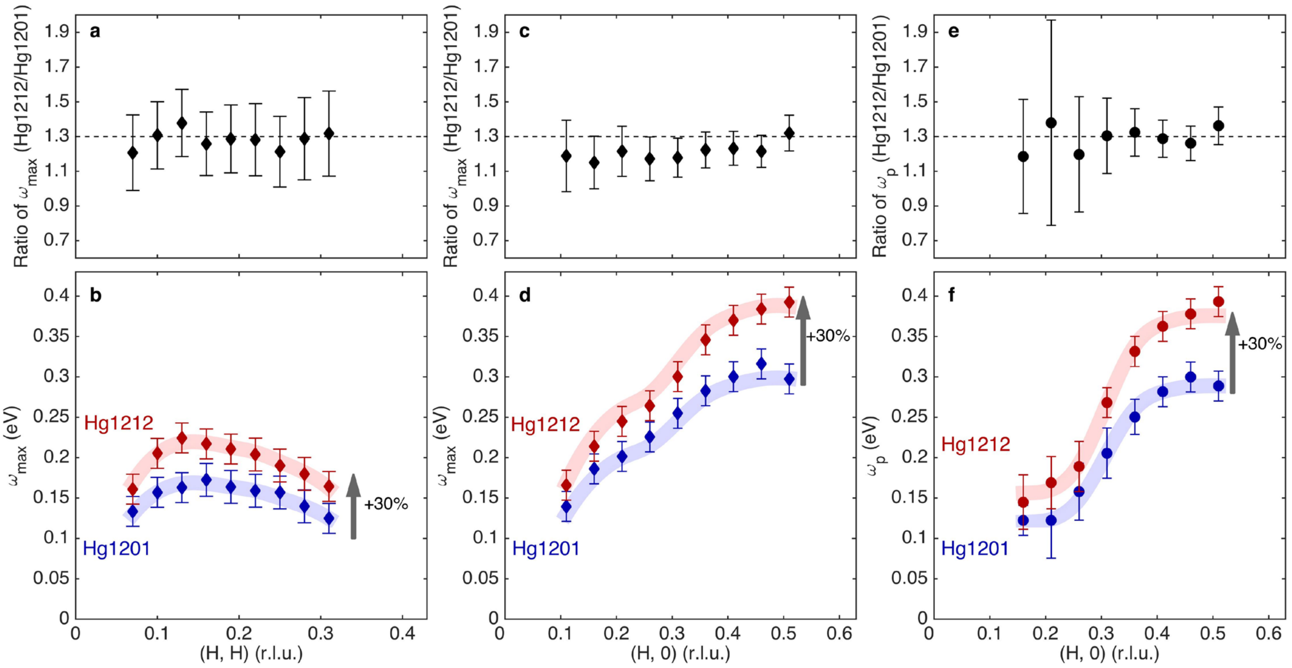

Supplementary Figure 6. ω_{max}, ω_p and their ratios between Hg1201 and Hg1212. a-b, The ratios of ω_{max} between Hg1201 and Hg1212 along $\mathbf{Q}_{//}$ = (H, H) and the estimated values of ω_{max}, respectively. The horizontal dashed line is a reference at 1.3, and the solid curves are guide to the eye with a 30% increase of ω_{max} from Hg1201 to Hg1212. **c-d**, same as **a-b**, but along (H, 0). **e-f**, same as **a-b**, but for ω_p along (H, 0). All the plotted values and uncertainties (error bars, 1 s.d.) are summarised in Supplementary Table 2.

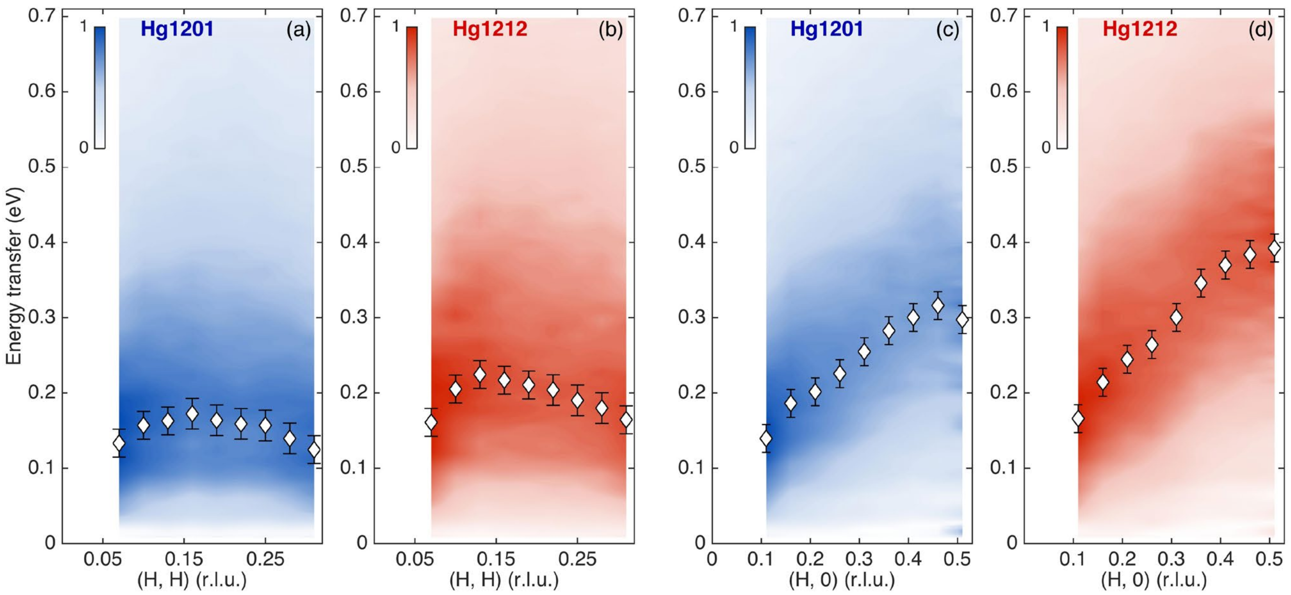

Supplementary Figure 7. The comparison of RIXS intensities arising from paramagnon. Same as Fig. 2, but in the same energy range.

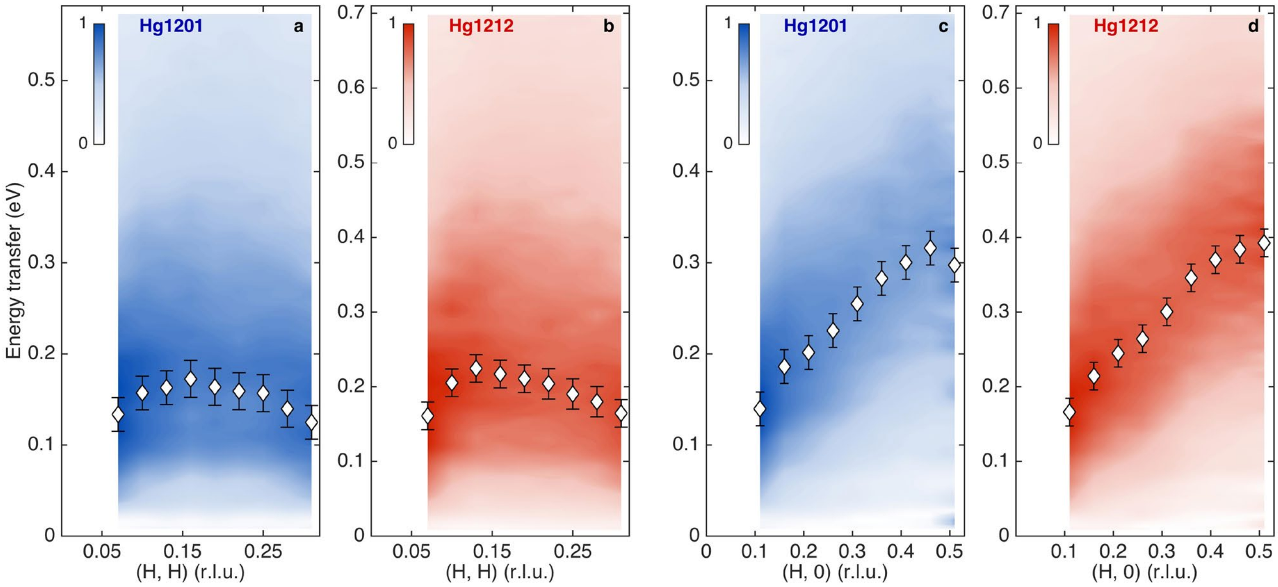

Supplementary Figure 8. Alternative comparison of paramagnon energy scales. Same as Fig. 2, but with the Hg1212 panels **b** and **d** having an energy scale 122% that of the Hg1201 panels **a** and **c**.

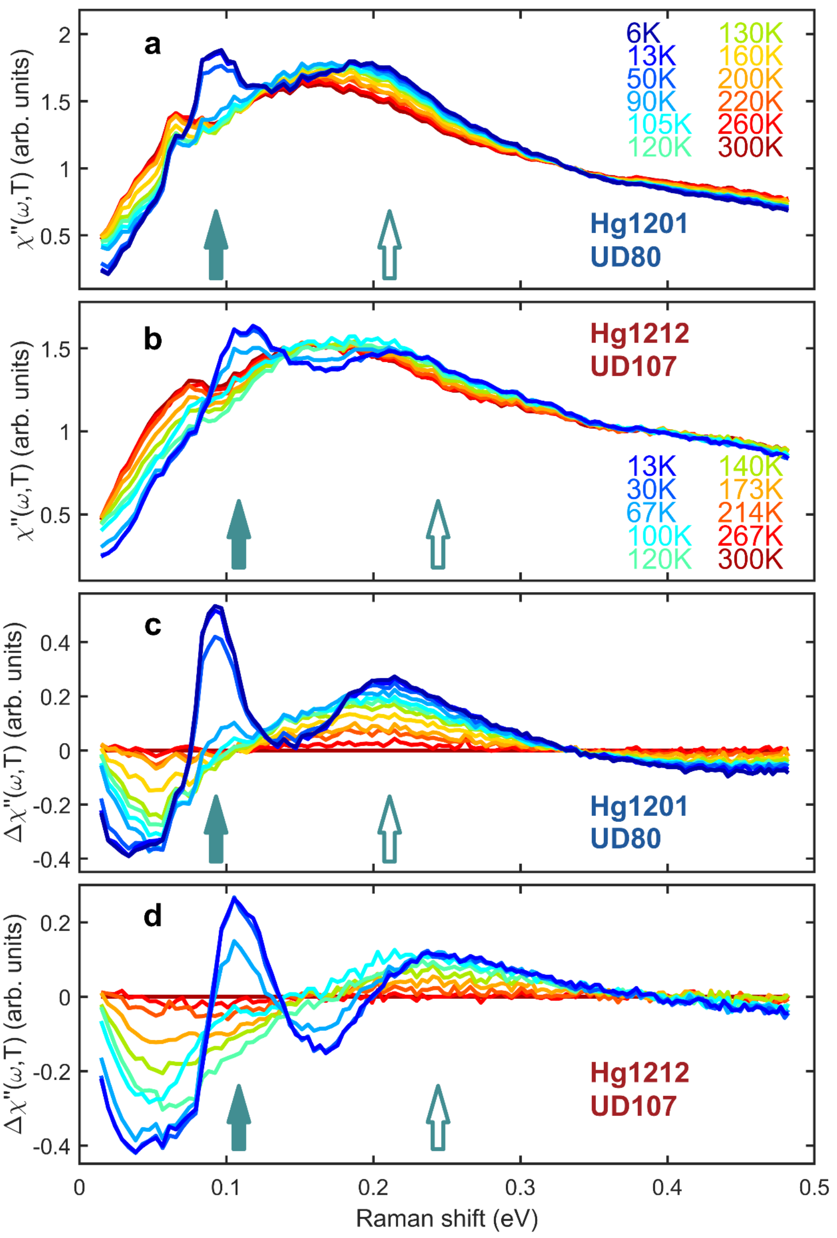

Supplementary Figure 9. Full data set of electronic Raman scattering spectra. a-b, Bose-factor-corrected B_{1g} spectra for Hg1201 and Hg1212, respectively. **c-d,** Spectral change relative to 300 K. Solid and empty arrows indicate the energies of the pair-breaking peak and the two-paramagnon peak at the lowest measured temperature, respectively.

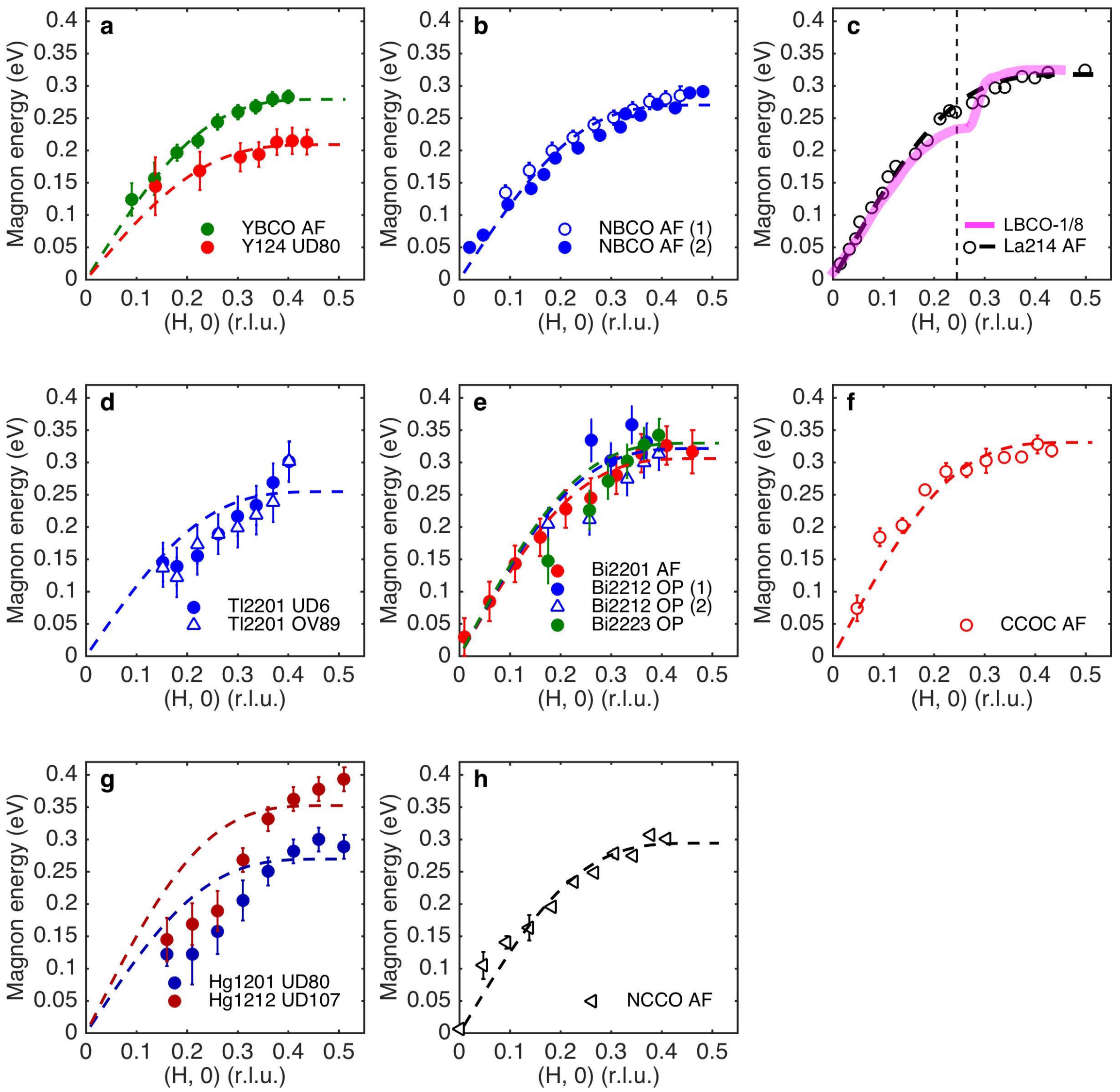

Supplementary Figure 10. Extraction of *J* for different cuprates. a-h, We consider the propagation energy ω_p, which is the same as ω_0 and ω_{max} for antiferromagnetic (AF) parent compounds. Sources of data and the results of the fitting (dashed curves) are summarised in Supplementary Table 3. For doped cuprates, only data points at $H \geq 0.3$ are considered for the fits. **c**, The solid curve in mageta is the paramagnon dispersion of LBCO-1/8 extracted from Ref. [1], which exhibits the energy anomaly at Q_{CDW} labeled by vertical dashed line.

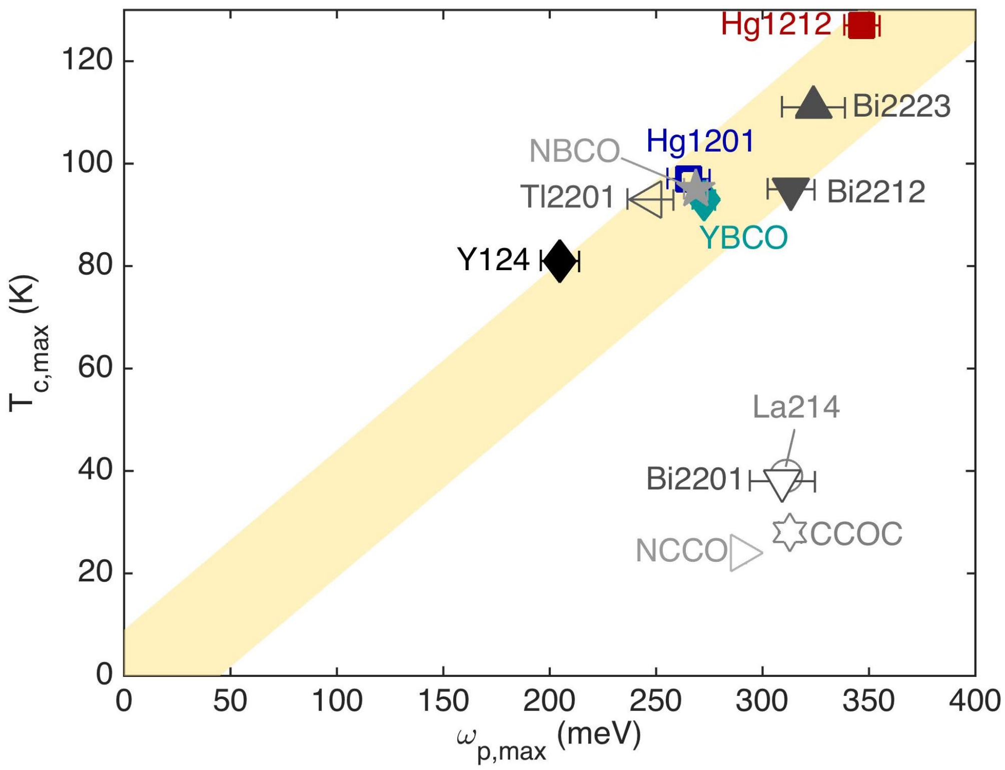

Supplementary Figure 11. $T_{c,max}$ versus $\omega_{p,max}$ in different cuprates. $\omega_{p,max}$ is the averaged propagation frequency close to the zone corner ($Q_{//}$ = (0.30, 0) and above). All values are determined from in Supplementary Fig. 10 and listed in Supplementary Table 4. Solid line is a guide to the eye.

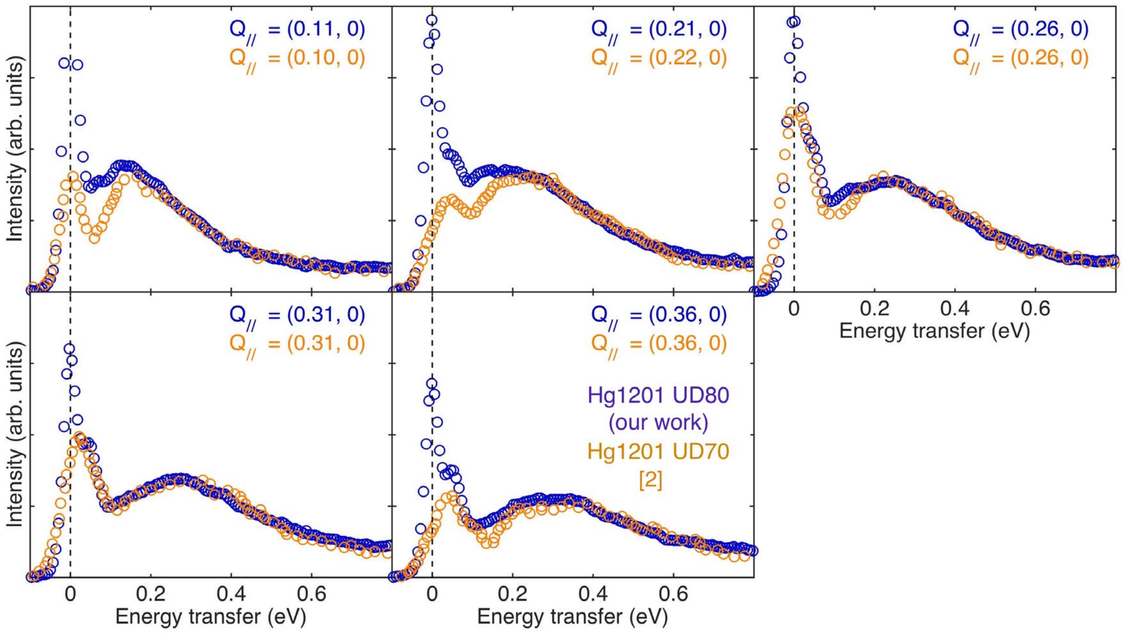

Supplementary Figure 12. Comparison of our RIXS spectra for Hg1201 with published data. Blue circles are our data on Hg1201 UD80 taken with the π–polarised incident x-rays and an overall energy resolution of 37 meV. Orange circles are data digitalized from the supplemental materials for Ref. [2] taken on a crystal of somewhat lower doping (UD70), with π–polarised incident x-rays and 60 meV energy resolution. The two data sets are normalised by the intensity at around 0.8 eV. Despite the different manifestations of the elastic and single-phonon intensities which are partly affected by the difference in the energy resolution, the broad paramagnon peaks are highly consistent between the two data sets.

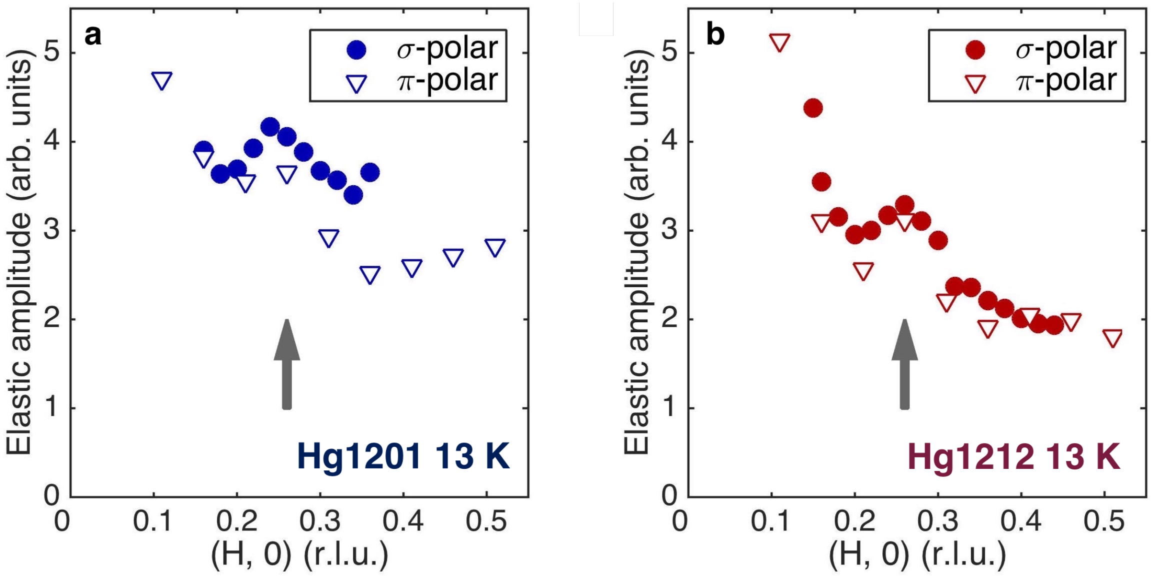

Supplementary Figure 13. Signature for short-range charge order in our Hg1201 and Hg1212 samples. a, Filled circles and empty triangles are elastic-intensity amplitudes measured with π- and σ-polarised incident *x*-rays, respectively, for Hg1201. The intensity maximum around $\mathbf{Q}_{//}$ = (0.26, 0) were observed with two different polarisations, indicative of charge order. **b,** Same as **a,** but for Hg1212. Arrows indicate H = 0.26.

$Q_{//}$ (H, K)	Hg1201				Hg1212			
	ω_{max}	ω_0	γ	ω_p	ω_{max}	ω_0	γ	ω_p
(0, 0.11)	140(19)	243(19)	243(23)		166(19)	305(19)	328(60)	
(0, 0.16)	186(19)	287(19)	257(21)	122(19)	214(19)	329(19)	285(31)	145(34)
(0, 0.21)	202(19)	317(19)	284(40)	123(47)	245(19)	361(19)	302(36)	169(32)
(0, 0.26)	226(19)	344(19)	294(40)	158(35)	264(19)	383(19)	314(37)	189(31)
(0, 0.31)	255(19)	373(21)	306(52)	206(31)	300(19)	411(19)	308(41)	268(19)
(0, 0.36)	283(19)	397(23)	306(53)	251(22)	346(19)	438(19)	282(30)	332(19)
(0, 0.41)	300(19)	415(26)	304(55)	282(19)	370(19)	454(19)	271(31)	363(19)
(0, 0.46)	316(19)	431(30)	309(63)	300(19)	384(19)	461(19)	262(26)	378(19)
(0, 0.51)	298(19)	399(19)	275(37)	289(19)	393(19)	464(19)	245(27)	393(19)
(0.07, 0.07)	133(19)	270(19)	314(40)		161(19)	306(19)	339(66)	
(0.10, 0.10)	157(19)	297(19)	322(35)		205(19)	337(19)	320(44)	
(0.13, 0.13)	163(19)	317(19)	355(68)		225(19)	363(19)	338(49)	
(0.16, 0.16)	173(20)	341(21)	384(74)		217(19)	383(22)	391(65)	
(0.19, 0.19)	164(20)	344(28)	415(98)		211(19)	398(32)	438(96)	
(0.22, 0.22)	159(20)	357(33)	448(116)		204(20)	406(38)	464(113)	
(0.25, 0.25)	157(20)	350(34)	445(119)		190(20)	413(43)	501(132)	
(0.28, 0.28)	140(20)	336(34)	449(126)		180(20)	404(45)	504(139)	
(0.31, 0.31)	125(19)	336(38)	504(136)		164(19)	380(49)	503(146)	

Supplementary Table 1. Fitting parameters for the paramagnon RIXS component in Hg1201 and Hg1212. Best-fit estimates of ω_{max}, $\omega_{0,}$ γ and ω_p are followed by uncertainties (1 s.d.) in parentheses, all in units of meV. Values of $\mathbf{Q}_{//}$ are in units of r.l.u.

$Q_{//}$ (H, K)	Increment from Hg1201 to Hg1212 (%)				
	ω_{max}	ω_p	$\omega_{p,max}$	J	$T_{c,max}$
(0, 0.11)	19(21)		**31(6)**	**30(11)**	**31**
(0, 0.16)	15(16)	19(33)			
(0, 0.21)	22(14)	38(59)			
(0, 0.26)	17(13)	20(33)			
(0, 0.31)	18(11)	30(22)			
(0, 0.36)	22(10)	32(14)			
(0, 0.41)	23(10)	29(11)			
(0, 0.46)	22(9)	26(10)			
(0, 0.51)	32(10)	36(11)			
(0.07, 0.07)	21(22)				
(0.10, 0.10)	31(19)				
(0.13, 0.13)	38(19)				
(0.16, 0.16)	26(18)				
(0.19, 0.19)	29(20)				
(0.22, 0.22)	28(21)				
(0.25, 0.25)	21(20)				
(0.28, 0.28)	29(24)				
(0.31 0.31)	32(25)				

Supplementary Table 2. Percentage increase in $T_{c,max}$, J, $\omega_{p,max}$, ω_p and ω_{max} from Hg1201 to Hg1212. The values (error bars, 1 s.d.) are calculated from the data in Supplementary Tables 1 and 4.

Crystal system	Tetragonal			
Compound	**$HgBa_2CuO_{4+\delta}$ [3]**	**$HgBa_2CaCu_2O_{6+\delta}$ [4]**	**$HgBa_2Ca_2Cu_3O_{10+\delta}$ [5]**	**Disorder type**
Cu-O-Cu angle (°)	180.0	179.4	178.4	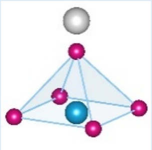
Apical Distance (Å)	2.786	2.775	2.741	
$T_{c\ max}$ (K)	97	127	135	
Space group	P4/mmm	P4/mmm	P4/mmm	
Crystal system	Orthorhombic	Tetragonal		
Compound	**$Tl_2Ba_2CuO_{6+\delta}$ [6]**	**$Tl_2Ba_2CaCu_2O_{8+\delta}$ [7]**	**$Tl_2Ba_2Ca_2Cu_3O_{10+\delta}$ [8]**	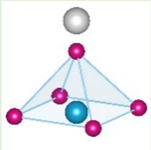
Cu-O-Cu angle (°)	180.0	178.4	179.4	
Apical Distance (Å)	2.717	2.699	2.680	
$T_{c\ max}$ (K)	93	112	127	
Space group	Fmmm	I4/mmm	I4/mmm	
Crystal system	Orthorhombic			
Compound	**$Bi_2Sr_{2-x}La_xCuO_{6+\delta}$ [9]**	**$Bi_{2+x}Sr_{2-x}CaCu_2O_{8+\delta}$ [10]**	**$Bi_{2+x}Sr_{2-x}Ca_2Cu_3O_{10+\delta}$ [11]**	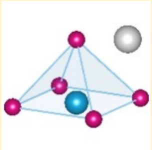
Cu-O-Cu angle (°)	174.1	162.7/178.6	160.8 / 176.8	
Apical Distance (Å)	2.589	2.432	2.201	
$T_{c\ max}$ (K)	38	95	111	
Space group	Cccm	Ccc2	Ccc2	
Crystal system	Orthorhombic			
Compound		**$NdBa_2Cu_3O_{6+\delta}$ [12]**		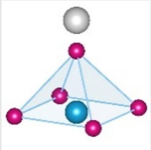
Cu-O-Cu angle (°)		163.4/165.6		
Apical Distance (Å)		2.207		
$T_{c\ max}$ (K)		95		
Space group		Pmmm		
Crystal system	Orthorhombic			
Compound		**$YBa_2Cu_3O_{6+\delta}$ [13]**		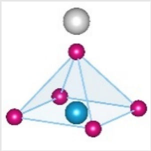
Cu-O-Cu angle (°)		165.3/166.3		
Apical Distance (Å)		2.341		
$T_{c\ max}$ (K)		93		
Space group		Pmmm		
Crystal system	Orthorhombic			
Compound		**$YBa_2Cu_4O_8$ [14]**		N.A.
Cu-O-Cu angle (°)		165.2/165.7		
Apical Distance (Å)		2.294		
$T_{c\ max}$ (K)		81		
Space group		Cmmm		

Crystal system	Tetragonal /Orthorhombic	Orthorhombic		
Compound	**$La_{2-x}Sr_xCuO_4$ [15]**	**$La_{2-x}Sr_xCaCu_2O_{6+\delta}$ [16]**		**Disorder type**
Cu-O-Cu angle (°)	176.4 / 180.0	176.0		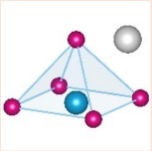
Apical Distance (Å)	2.414 / 2.413	2.310		
$T_{c\ max}$ (K)	39	60		
Space group	Cmca / I4/mmm	I4/mmm		
Crystal system	Tetragonal			
Compound	**$La_{2-x}Ba_xCuO_4$ [17]**	**$La_{2-x}Ba_xCaCu_2O_{6+\delta}$ [18]**		**Disorder type**
Cu-O-Cu angle (°)	180.0	176.264		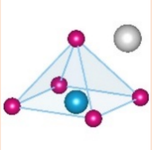
Apical Distance (Å)	2.429	2.303		
$T_{c\ max}$ (K)	33	47		
Space group	I4/mmm	I4/mmm		
Crystal system	Tetragonal			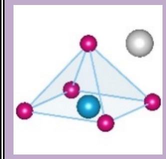
Compound	**$Ca_{2-x}Na_xCuO_2Cl_2$ [19]**			
Cu-O-Cu angle (°)	180.0			
Apical Distance (Å)	2.753			
$T_{c,max}$ (K)	28			
Space group	I4/mmm			
Crystal system	Tetragonal			N.A.
Compound	**$Nd_{2-x}Ce_xCuO_4$ [20]**			
Cu-O-Cu angle (°)	180.0			
Apical Distance (Å)	n.a			
$T_{c,max}$ (K)	24			
Space group	I4/mmm			
Note: The structure information was obtained from the reference-linked database (ICSD data and Pearson's data).				

Supplementary Table 3. Detailed structural information for different cuprates. Materials belonging to the same family, *i.e.*, as different Ruddlesden-Popper members, are classified by the same colour. Cu-O-Cu angles for the triple-layer cuprates refer to the outer CuO_2 layers (the Cu-O-Cu angle of the inner CuO_2 layer is 180°) [5]. The optimally doped $La_{2-x}Sr_xCuO_4$ has two crystallographic phases: the low-temperature orthorhombic structure (Cmca) and the high-temperature tetragonal structure (I4/mmm) [15], and the corresponding Cu-O-Cu angle is 176.4° and 180.0° respectively. The space group of Bi2212 was reported to be either centrosymmetric Cccm or its non-centrosymmetric subgroup Ccc2 [10], but a very recent report [21] with higher accuracy supports Ccc2 space group. Therefore, two different Cu-O-Cu angles for Bi2212 exists due to its lower symmetry than Bi2201, and their average is smaller than that in Bi2201, *i.e.*, with further away from

being a straight Cu-O-Cu bond. Bi2223 is similar to Bi2212. The illustration of chemical disorder relative to the CuO_5 pyramids or the CuO_6 octahedra is after Ref. [22].

**Compound**	**Reference**	$T_{c,max}$ (K)	J (meV)	$\omega_{p,max}$ (meV)
La214	[23]	39	157(1)	311(4)
CCOC	[24]	28	166(4)	313(5)
YBCO	[25]	93	140(2)	272(5)
Y124	[26]	81	105(2)	205(9)
NBCO	[26, 27]	95	135(2)	269(6)
Tl2201	[25]	93	127(7)	247(11)
Bi2201	[28]	38	153(3)	309(15)
Bi2212	[29, 30]	95	161(7)	313(11)
Bi2223	[29]	111	165(6)	324(15)
NCCO	[31]	24	147(4)	290(2)
Hg1201	this work	97	135(8)	265(10)
Hg1212	this work	127	176(11)	347(8)

Supplementary Table 4. $T_{c,max}$, J and $\omega_{p,max}$ of different cuprates. $\omega_{p,max}$ is the averaged propagation energy close to the zone corner (from $Q_{//}$ = (0.30, 0) to $Q_{//}$ = (0.50, 0)). $\omega_{p,max}$ and J are determined from data and fits in Supplementary Fig. 11.

Family	T_{pair} (K)	Doping	Technique	Reference
Bi2201	100	underdoped	Nernst effect	[32]
	90	underdoped	Torque Magnetization[1]	[33]
	120	underdoped	ARPES	[34]
	42	underdoped	Specific heat	[35]
	55	underdoped	Torque Magnetization [2]	[36]
Bi2212	130	underdoped	Nernst effect	[37]
	130	underdoped	Torque Magnetization[1]	[38]
	140	optimal	STM	[39]
	150	underdoped	ARPES	[40]
Bi2223	135	optimal	Nernst effect	[37]
La214	130	underdoped	Nernst effect	[37]
	120	underdoped	Torque Magnetization[1]	[33]
	48	optimal	Terahertz	[41]
	47	underdoped	Torque Magnetization [2]	[36]
YBCO	150	underdoped	Nernst effect	[42]
	130	optimal	Torque Magnetization[1]	[33]
	96	overdoped	Microwave	[43]
NBCO	130	underdoped	Infrared spectroscopy	[44]
Hg1201	105	underdoped	Microwave	[45]
	117	underdoped	Torque Magnetization [2]	[36]

Supplementary Table 5. T_{pair} of different cuprates measured by different techniques. As is shown in red font, the ARPES results on Bi2201 [34] and Bi2212 [40] indicated that their T_{pair} are around 120-150 K, consistent with previous works in the Bi-family, YBCO, La214 and NBCO determined from Nernst effect [32, 37, 42], torque magnetization [33, 38], scanning tunnelling microscopy (STM) [39] and infrared spectroscopy [44]. However, other results (in blue font) by Microwave [43, 45], terahertz conductivity [41], specific heat [35] and the different interpretation of torque magnetometry data [36] suggested that the signatures of superconducting fluctuations exist only in a relatively narrow temperature range above T_c.

[1, 2] the same technique but with different analytical methods.

References

1. Miao, H., et al., *High-temperature charge density wave correlations in $La_{1.875}Ba_{0.125}CuO_4$ without spin–charge locking.* Proceedings of the National Academy of Sciences, 2017. **114**(47): p. 12430-12435.
2. Yu, B., et al., *Unusual Dynamic Charge Correlations in Simple-Tetragonal $HgBa_2CuO_{4+\delta}$.* Physical Review X, 2020. **10**(2): p. 021059.
3. Huang, Q., et al., *Oxygen dependence of the crystal structure of $HgBa_2CuO_{4+\delta}$ and its relation to superconductivity.* Physical Review B, 1995. **52**(1): p. 462-470.
4. Radaelli, P.G., et al., *Structure, doping and superconductivity in $HgBa_2CaCu_2O_{6+\delta}$ ($T_c \leqslant 128$ K).* Physica C: Superconductivity, 1993. **216**(1-2): p. 29-35.
5. Wagner, J.L., et al., *Structure and superconductivity of $HgBa_2Ca_2Cu_3O_{8+\delta}$.* Physical Review B, 1995. **51**(21): p. 15407-15414.
6. Wagner, J.L., et al., *Multiple defects in overdoped $Tl_2Ba_2CuO_{6+\delta}$: effects on structure and superconductivity.* Physica C: Superconductivity, 1997. **277**(3-4): p. 170-182.
7. Subramanian, M.A., et al., *Crystal structure of the high-temperature superconductor $Tl_2Ba_2CaCu_2O_8$.* Nature, 1988. **332**(6163): p. 420-422.
8. Sinclair, D.C., et al., *Cation distribution and composition of the Tl-2223 superconductor from combined powder neutron and resonant X-ray diffraction.* Physica C: Superconductivity, 1994. **225**(3-4): p. 307-316.
9. Torardi, C.C., et al., *Structures of the superconducting oxides $Tl_2Ba_2CuO_6$ and $Bi_2Sr_2CuO_6$.* Physical Review B, 1988. **38**(1): p. 225-231.
10. Gladyshevskii, R.E. and R. Flükiger, *Modulated structure of $Bi_2Sr_2CaCu_2O_{8+\delta}$, a high-Tc superconductor with monoclinic symmetry.* Acta Crystallographica Section B Structural Science, 1996. **52**(1): p. 38-53.
11. Shamray, V.F., A.B. Mikhailova, and A.V. Mitin, *Crystal structure and superconductivity of Bi-2223.* Crystallography Reports, 2009. **54**(4): p. 584-590.
12. Takita, K., et al., *X-Ray Diffraction Study on the Crystal Structure of $Nd_{1+x}Ba_{2-x}Cu_3O_{7-\delta}$.* Japanese Journal of Applied Physics, 1988. **27**(1A): p. L57-L60.
13. Calestani, G. and C. Rizzoli, *Crystal structure of the $YBa_2Cu_3O_7$ superconductor by single-crystal X-ray diffraction.* Nature, 1987. **328**(6131): p. 606-607.
14. Lightfoot, P., et al., *Redetermination of the structure of the 80 K superconductor $YBa_2Cu_4O_8$ by time-of-flight neutron powder diffraction.* Acta Crystallographica Section C Crystal Structure Communications, 1991. **47**(6): p. 1143-1145.
15. Radaelli, P.G., et al., *Structural and superconducting properties of $La_{2-x}Sr_xCuO_4$ as a function of Sr content.* Physical Review B, 1994. **49**(6): p. 4163-4175.
16. Shaked, H., et al., *Defect structure and superconducting properties of $La_{1.8}Sr_xCa_{1.2-x}Cu_2O_{6-\delta}$.* Physical Review B, 1993. **48**(17): p. 12941-12950.
17. Jorgensen, J.D., et al., *Lattice instability and high-Tc superconductivity in $La_{2-x}Ba_xCuO_4$.* Physical Review Letters, 1987. **58**(10): p. 1024-1027.
18. Navarro, J.M., et al., *Crystal structure of $La_{1.85}Ba_{0.15}CaCu_2O_{6+y}$ determined by neutron powder diffraction.* Solid State Communications, 1992. **81**(8): p. 677-681.
19. Argyriou, D.N., et al., *Structure and superconductivity without apical oxygens in $(Ca,Na)_2CuO_2Cl_2$.* Physical Review B, 1995. **51**(13): p. 8434-8437.

20. Takagi, H., S. Uchida, and Y. Tokura, *Superconductivity produced by electron doping in CuO_2-layered compounds.* Physical Review Letters, 1989. **62**(10): p. 1197-1200.
21. Ivanov, A.A., et al., *Local Noncentrosymmetric Structure of $Bi_2Sr_2CaCu_2O_{8+y}$ by X-ray Magnetic Circular Dichroism at Cu K-Edge XANES.* Journal of Superconductivity and Novel Magnetism, 2017. **31**(3): p. 663-670.
22. Eisaki, H., et al., *Effect of chemical inhomogeneity in bismuth-based copper oxide superconductors.* Physical Review B, 2004. **69**(6): p. 064512.
23. Headings, N.S., et al., *Anomalous high-energy spin excitations in the high-T_c superconductor-parent antiferromagnet La_2CuO_4.* Physical Review Letters, 2010. **105**(24): p. 247001.
24. Lebert, B.W., et al., *Resonant inelastic x-ray scattering study of spin-wave excitations in the cuprate parent compound $Ca_2CuO_2Cl_2$.* Physical Review B, 2017. **95**(15): p. 155110.
25. Le Tacon, M., et al., *Dispersive spin excitations in highly overdoped cuprates revealed by resonant inelastic x-ray scattering.* Physical Review B, 2013. **88**(2): p. 020501.
26. Le Tacon, M., et al., *Intense paramagnon excitations in a large family of high-temperature superconductors.* Nature Physics, 2011. **7**(9): p. 725-730.
27. Peng, Y.Y., et al., *Influence of apical oxygen on the extent of in-plane exchange interaction in cuprate superconductors.* Nature Physics, 2017. **13**(12): p. 1201-1206.
28. Peng, Y.Y., et al., *Dispersion, damping, and intensity of spin excitations in the monolayer $(Bi,Pb)_2(Sr,La)_2CuO_{6+\delta}$ cuprate superconductor family.* Physical Review B, 2018. **98**(14): p. 144507.
29. Dean, M.P.M., et al., *Itinerant effects and enhanced magnetic interactions in Bi-based multilayer cuprates.* Physical Review B, 2014. **90**(22): p. 220506.
30. Peng, Y.Y., et al., *Magnetic excitations and phonons simultaneously studied by resonant inelastic x-ray scattering in optimally doped $Bi_{1.5}Pb_{0.55}Sr_{1.6}La_{0.4}CuO_{6+\delta}$.* Physical Review B, 2015. **92**(6): p. 064517.
31. Ishii, K., et al., *High-energy spin and charge excitations in electron-doped copper oxide superconductors.* Nature Communications, 2014. **5**(1): p. 3714.
32. Wang, Y., et al., *Onset of the vortexlike Nernst signal above T_c in $La_{2-x}Sr_xCuO_4$ and $Bi_2Sr_{2-y}La_yCuO_6$.* Physical Review B, 2001. **64**(22): p. 224519.
33. Li, L., et al., *Diamagnetism and Cooper pairing above T_c in cuprates.* Physical Review B, 2010. **81**(5): p. 054510.
34. Kondo, T., et al., *Disentangling Cooper-pair formation above the transition temperature from the pseudogap state in the cuprates.* Nature Physics, 2010. **7**(1): p. 21-25.
35. Wen, H.-H., et al., *Specific-Heat Measurement of a Residual Superconducting State in the Normal State of Underdoped $Bi_2Sr_{2-x}La_xCuO_{6+\delta}$ Cuprate Superconductors.* Physical Review Letters, 2009. **103**(6): p. 067002.
36. Yu, G., et al., *Universal precursor of superconductivity in the cuprates.* Physical Review B, 2019. **99**(21): p. 214502.
37. Wang, Y., L. Li, and N.P. Ong, *Nernst effect in high-Tcsuperconductors.* Physical Review B, 2006. **73**(2): p. 024510.
38. Wang, Y., et al., *Field-Enhanced Diamagnetism in the Pseudogap State of the Cuprate $Bi_2Sr_2CaCu_2O_{8+\delta}$ Superconductor in an Intense Magnetic Field.* Physical Review Letters, 2005. **95**(24): p. 247002.
39. Gomes, K.K., et al., *Visualizing pair formation on the atomic scale in the high-T_c superconductor $Bi_2Sr_2CaCu_2O_{8+\delta}$.* Nature, 2007. **447**(7144): p. 569-572.

40. Zhang, W., et al., *Signatures of superconductivity and pseudogap formation in nonequilibrium nodal quasiparticles revealed by ultrafast angle-resolved photoemission.* Physical Review B, 2013. **88**(24): p. 245132.

41. Bilbro, L.S., et al., *Temporal correlations of superconductivity above the transition temperature in* $La_{2-x}Sr_xCuO_4$ *probed by terahertz spectroscopy.* Nature Physics, 2011. **7**(4): p. 298-302.

42. Ong, N.P., et al., *Vorticity and the Nernst effect in cuprate superconductors.* Annalen der Physik, 2004. **13**(12): p. 9-14.

43. Grbić, M.S., et al., *Temperature range of superconducting fluctuations above* T_c *in* $YBa_2Cu_3O_{7-\delta}$ *single crystals.* Physical Review B, 2011. **83**(14): p. 144508.

44. Baldini, E., et al., *Clocking the onset of bilayer coherence in a high-*T_c *cuprate.* Physical Review B, 2017. **95**(2): p. 024501.

45. Grbić, M.S., et al., *Microwave measurements of the in-plane andc-axis conductivity in* $HgBa_2CuO_{4+\delta}$*: Discriminating between superconducting fluctuations and pseudogap effects.* Physical Review B, 2009. **80**(9): p. 094511.
